# Association of the dietary index for gut microbiota and chronic obstructive pulmonary disease: a cross-sectional study

**DOI:** 10.3389/fnut.2025.1596424

**Published:** 2025-08-26

**Authors:** Ting Ao, Yingxiu Huang, Peng Zhen, Ming Hu

**Affiliations:** Department of Infectious Diseases, Beijing Luhe Hospital, Capital Medical University, Beijing, China

**Keywords:** dietary index for gut microbiota, chronic obstructive pulmonary disease, NHANES, dietary, gut microbiota

## Abstract

**Background:**

Gut microbiota plays a vital role in maintaining human health. The dietary index for gut microbiota (DI-GM), which reflects the diversity of gut microbiota, has not yet been studied for chronic obstructive pulmonary disease (COPD).

**Methods:**

This cross-sectional study analyzed data from adult participants in the 1999–2018 National Health and Nutrition Examination Survey. COPD was identified based on self-reported data. The DI-GM was calculated using dietary recall data. Multivariable logistic regression models were employed to assess the association between DI-GM and COPD. To evaluate the robustness of the association, subgroup and sensitivity analyses were applied.

**Results:**

Increased DI-GM was associated with a decreased prevalence of COPD (OR = 0.96; 95% CI, 0.92–0.99; *P* = 0.016). Greater beneficial gut microbiota scores were inversely related to the prevalence of COPD (OR = 0.95; 95% CI, 0.91–1.00; *P* = 0.03). Both unweighted and multiple interpolated logistic regression analyses confirmed that the relationship remained consistent. Subgroup analyses further supported the robustness of the findings.

**Conclusion:**

A negative association was observed between DI-GM and the prevalence of COPD.

## 1 Introduction

Chronic obstructive pulmonary disease (COPD) is a multifaceted lung disorder marked by chronic respiratory symptoms, such as dyspnea, cough, expectoration, and/or exacerbations. These symptoms result from abnormalities in the airways (including bronchitis and bronchiolitis) and/or the alveoli (such as emphysema), which lead to chronic and often progressive airflow limitation (1). According to the Global Burden of Diseases, Injuries, and Risk Factors Study 2021, COPD is the fourth most common cause of death globally (2). The rising incidence and significant socioeconomic burden it imposes on societies have made it an escalating public health issue. Although there have been improvements in managing symptoms and preventing acute exacerbations, limited progress has been made in slowing disease progression or reducing mortality (3). Therefore, it is of utmost importance to discover modifiable risk factors that are responsible for both the initiation and progression of the disease.

A wide range of research has identified an association between gut microbiota and COPD, suggesting that gut microbiota may offer a promising target for COPD prevention and treatment (4, 5). Additionally, a growing body of evidence indicates that dietary patterns significantly influence gut microbiota composition (6). As a result, dietary interventions are increasingly recognized as a modifiable risk factor for COPD (7, 8). Kase et al. conducted a comprehensive review of 106 articles examining the relationship between gut microbiota and diet in adults, identifying 14 dietary components that either promote or hinder gut microbiota health. Based on these findings, they created a new dietary index for gut microbiota (DI-GM) to evaluate the impact of diets on gut microbiota health (9). Furthermore, DI-GM was found to be positively associated with urinary enterodiol and enterolactone, biomarkers indicative of gut microbiota diversity, underscoring the relationship between the index and the diversity of gut microbiota. Hence, the DI-GM serves as an effective tool for identifying dietary patterns that either promote or detract from gut microbiota health. It could be a valuable standardized method for evaluating a balanced diet aimed at promoting gut microbiota health. In addition, the DI-GM offers opportunities for fostering collaboration between diverse fields, including microbiology, nutrition, medicine, and epidemiology.

Given accumulating evidence on the role of gut microbiota in the development of COPD, investigating the association between DI-GM and COPD may yield new insights into potential dietary prevention strategies. Thus, this study aimed to use adult data from the National Health and Nutrition Examination Survey (NHANES) to explore the relationship between DI-GM and COPD.

## 2 Material and methods

### 2.1 Study population

The NHANES is an ongoing survey that measures the health and nutrition of adults and children in the United States. It employs sophisticated multistage probability cluster designs for data collection and study methodology, ensuring the gathering of precise and comprehensive data. The NHANES project was approved by the Research Ethics Committee of the National Center for Health Statistics (NCHS), with informed consent obtained from all participants. Additional details are available on the NCHS website. The secondary analysis conducted for this study did not require further Institutional Review Board approval.

**Figure 1:**
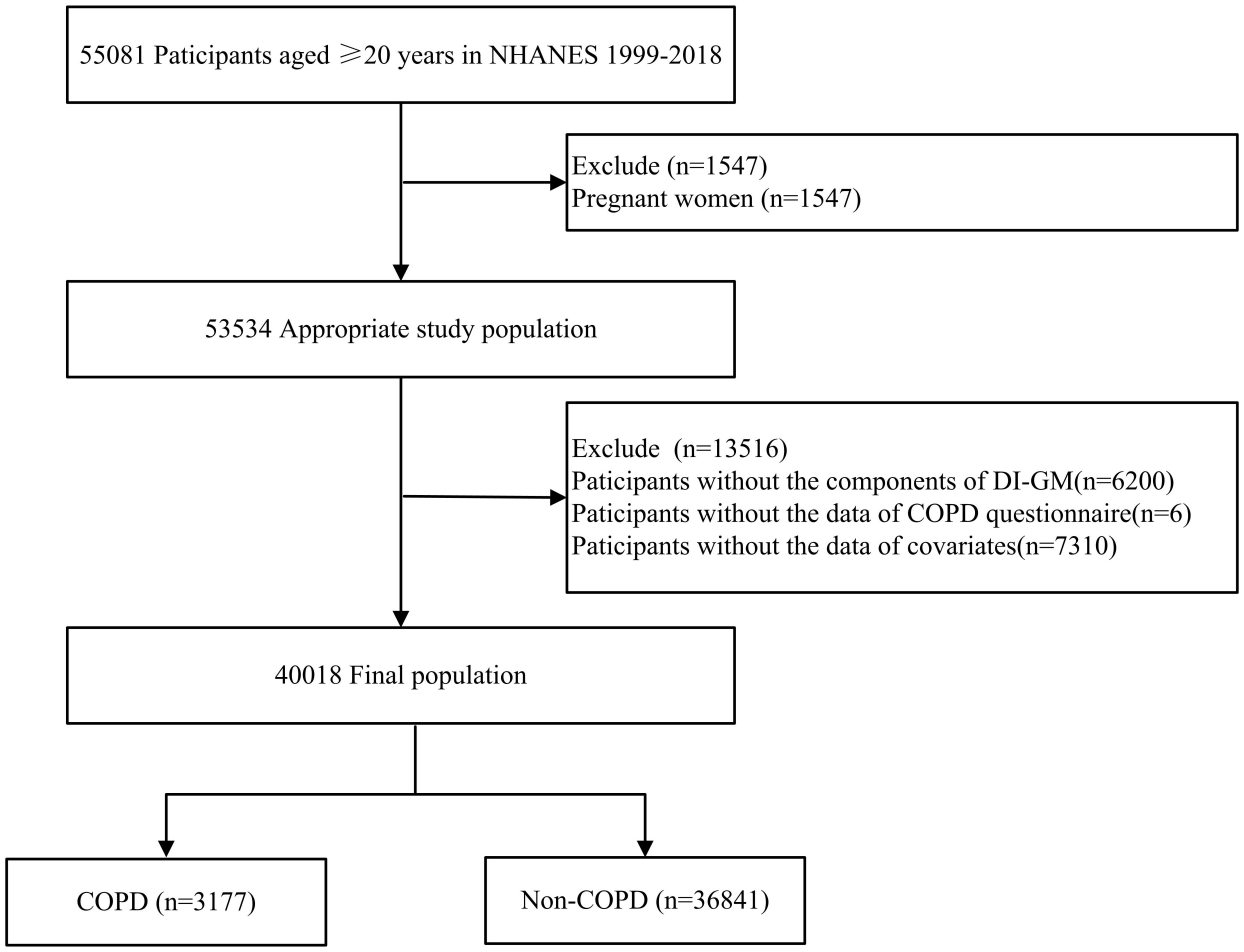
Flow chart of patient selection. NHANES, national health and nutrition examination survey; COPD, chronic obstructive pulmonary disease; DI-GM, dietary index for gut microbiota.

This study was a retrospective analysis using data from NHANES (1999–2018), which included 55,081 participants aged 20 years and older. Exclusion criteria for individuals included pregnant (*n* = 1,547), absence of DI-GM components (*n* = 6,200), missing COPD survey data (*n* = 6), or missing covariates data (*n* = 7,310), such as marital status, poverty income ratio (PIR), body mass index (BMI), educational level, drinking and smoking status, cardiovascular disease (CVD), hypertension, diabetes, and hyperlipidemia (Figure 1).

### 2.2 COPD

COPD was defined based on responses to the clinical questions “Has a doctor ever said you had COPD”, “Have you ever been told you had chronic bronchitis” or “Have you ever been told you had emphysema” from the medical conditions questionnaire, as validated in a previous study (10).

### 2.3 Assessment of dietary index for gut microbiota

According to the scoring criteria established by Kase et al., the DI-GM was composed of 14 specific food items or nutrients (Supplementary Table 1). These included beneficial items such as avocado, broccoli, soybean, chickpeas, cranberries, fermented dairy, coffee, green tea (data unavailable in NHANES due to the lack of specific tea information), whole grains, and fiber. In contrast, refined grains, red meat, processed meat, and diets with ≥40% of energy from fat were categorized as adverse components (9). The DI-GM was calculated using dietary recall data from the NHANES dataset (1999–2018). Individuals whose consumption was above the sex-specific median for beneficial components or below the median for unfavorable components were assigned a score of 1. In contrast, a score of 0 was given to individuals whose consumption was below the sex-specific median for beneficial components or above the median for unfavorable components. The individual component scores were then summed to derive the overall DI-GM score, which ranged from 0 to 13 (with scores from 0 to 9 for beneficial to gut microbiota and 0 to 4 for unfavorable to gut microbiota). A higher DI-GM score indicates a more favorable gut microbiota. In this study, the DI-GM was categorized into groups according to 0–3, 4, 5, and ≥6 (11).

### 2.4 Covariates

All data were gathered and documented by investigators who received uniform training. Informed by existing research and clinical expertise, several potential confounding factors were considered, such as age, sex, race/ethnicity, education, PIR, marital status, smoking status, drinking status, BMI, hypertension, diabetes, CVD, and hyperlipidemia (11, 12).

Sex was categorized as female or male. Race/ethnicity was grouped as non-Hispanic White, non-Hispanic Black, other Hispanic, Mexican American, or other. Marital status was categorized into two groups: married or living with a partner, and living alone. Education levels were classified into three groups: above high school, high school or equivalent, and less than high school. PIR was grouped into three levels based on the values 1.30 and 3.50. Smoking status was classified into three categories: never smoked, current smoker, or former smoker. Drinking status was categorized into three groups: never, former, or current. BMI was calculated using the standard method based on weight and height.

CVD diagnosis was based on self-reported physician diagnoses obtained through a standardized questionnaire on medical conditions. The participants were asked, “Has a doctor or other health expert ever informed you that you have congestive heart failure/coronary heart disease/angina pectoris/myocardial Infarction/stroke?” individuals who responded with “yes” to any of these questions were considered to have CVD (13). Hypertension was diagnosed if the mean systolic blood pressure was ≥140 mmHg, or the mean diastolic blood pressure was ≥90 mmHg, or if the individual had ever used antihypertensive drugs or had been previously diagnosed with hypertension by a doctor or healthcare professional (14). Diabetes was diagnosed if any of the following conditions were met: a physician diagnosed the individual with diabetes; fasting glucose was ≥7.0 mmol/l; random blood glucose was ≥11.1 mmol/l; glycohemoglobin was ≥6.5%; 2-h oral glucose tolerance test blood glucose was ≥11.1 mmol/l; or the individual was using diabetes medication or insulin (15). Hyperlipidemia was defined as having any of the following: triglycerides ≥ 150 mg/dl, total cholesterol ≥ 200 mg/dl, low-density lipoprotein ≥ 130 mg/dl, or high-density lipoprotein ≤ 50 mg/dl in females and ≤ 40 mg/dl in males. Furthermore, individuals who indicated the use of lipid-lowering medications were classified as having hyperlipidemia (14).

### 2.5 Statistical analysis

The characteristics of all individuals were summarized based on the presence or absence of COPD. Categorical variables were described by proportions (%) whereas continuous variables were presented as mean with standard deviation (SD) or medians with interquartile range (IQR), depending on the data distribution. Depending on the normality of the distribution, either the independent samples Student's *t*-test or Mann-Whitney *U*-test was used to compare the continuous data among groups. Chi-square or Fisher's exact test was used to compare categorical data, as appropriate.

Following the NHANES analysis guidelines, this analysis accounted for complex sampling designs and sampling weights (16). The analysis included the following variables: masked variance pseudo-cluster (SDMVPSU), masked variance pseudo-stratum (SDMVSTRA), and dietary weights. Specifically, dietary day one 4-year sample weight (WTDR4YR) was utilized, as the data from NHANES 1999–2000 and 2001–2002 were included. For the NHANES 2003–2018 data, dietary day one 2-year sample weight (WTDRD1) was applied. The sampling weights for the 1999–2018 period were calculated as follows: for the 1999–2002 data, the weights were 1/5 × WTDR4YR, while for other years, the weights were 1/10 × WTDRD1.

Multivariable weighted logistic regression models were employed to estimate the odds ratios (OR) and 95 percent confidence intervals (95% CIs) for the association between DI-GM and COPD. Four progressively adjusted models were used. Model 1 is severed as an unadjusted or crude model. Model 2 was adjusted for age, sex, and race/ethnicity. Model 3 was further adjusted for marital status, PIR, educational level, smoking status, drinking status, and BMI in addition to the variables in model 2. Model 4 included all variables from model 3 and additionally accounted for comorbidities, including CVD, hyperlipidemia, hypertension, and diabetes.

Furthermore, interaction and subgroup analyses were performed to evaluate the consistency of the relationship between DI-GM and COPD across various populations. These analyses were stratified by sex (male vs. female), age (20–60 vs. ≥60 year), BMI (<25, 25–30, or ≥30 kg/m^2^), CVD (yes vs. no), diabetes (yes vs. no), hyperlipidemia (yes vs. no), hypertension (yes vs. no), constipation status (yes vs. no), PIR groups (≤1.3, 1.3–3.5 or >3.5), and education levels (“above high school”, “high school or equivalent”, or “less than high school”).

Multiple sensitivity analyses were performed to examine the robustness of the results: (1) To address missing data, multiple imputations by chained equations were applied. Five imputed datasets were generated using the variables included in the final statistical model (17); (2) Multivariable unweighted logistic regression models were employed; (3) Based on model 4, we separately adjusted energy, protein, saturated fat, and dietary fiber intake to assess the association between the DI-GM and COPD; (4) we compared DI-GM and established indices such as alternative Mediterranean Diet Score (aMED) (18), Healthy Eating Index-2015 (HEI-2015) (19), and the Dietary Inflammatory Index (DII) (20) to assess their association with COPD. Receiver operating characteristic (ROC) curves were generated to assess the predictive performance for COPD.

All analyses were performed using R Statistical Software (Version 4.2.2, http://www.R-project.org, The R Foundation) and Free Statistics analysis platform (Version 2.0, Beijing, China). A *P*-value of < 0.05 in a two-sided test was regarded as statistically significant.

## 3 Results

### 3.1 Patients' characteristics

Table 1 presents the baseline characteristics of a sample representing 185.88 million U.S. adults, with an average age of 47.33 (16.89) years. Of these, 14.85 million were diagnosed with COPD. Individuals with COPD tended to be older, female, non-Hispanic White, and more likely to be smokers and drinkers. They also had higher BMI, and a greater incidence of CVD, hypertension, diabetes, and hyperlipidemia, as well as lower DI-GM (Table 1).

**Table 1:** Characteristics of the NHANES 1999–2018 participants.

**Patient characteristic**	**Total**	**Non-COPD**	**COPD**	***p*-value**
Weighted population, *n* (in millions)	185.88	171.03	14.85	
Age (years), Mean (SD)	47.33 (16.89)	46.70 (16.81)	54.68 (16.11)	<0.0001
**Sex**, ***n*** **(in millions), %**				<0.0001
Male	91.48 (49.21)	85.74 (50.13)	5.74 (38.64)	
Female	94.40 (50.79)	85.28 (49.87)	9.11 (61.36)	
**Race/ethnicity**, ***n*** **(in millions), %**				<0.0001
Non-Hispanic White	130.86 (70.40)	119.14 (69.66)	11.72 (78.90)	
Non-Hispanic Black	19.60 (10.54)	18.35 (10.73)	1.24 (8.38)	
Mexican American	14.26 (7.68)	13.79 (8.07)	0.47 (3.16)	
Other Hispanic	9.22 (4.97)	8.67 (5.07)	0.55 (3.72)	
Other	11.92 (6.42)	11.05 (6.47)	0.86 (5.84)	
**Marital status**, ***n*** **(in millions), %**				<0.0001
Married/Living with a partner	11.68 (62.86)	108.44 (63.41)	8.40 (56.56)	
Never married/Other	69.04 (37.14)	62.58 (36.59)	6.45 (43.44)	
**Poverty income ratio**, ***n*** **(in millions), %**				<0.0001
≤1.3	39.74 (21.38)	35.00 (20.47)	4.74 (31.92)	
1.3-3.5	65.75 (35.37)	59.86 (35.00)	5.89 (39.64)	
>3.5	80.39 (43.25)	76.16 (44.53)	4.22 (28.44)	
**Educational level**, ***n*** **(in millions), %**				<0.0001
Less than high school	29.54 (15.90)	26.25 (15.35)	3.28 (22.14)	
High school or equivalent	45.06 (24.24)	40.92 (23.93)	4.13 (27.85)	
Above high school	111.27 (59.86)	103.84 (60.72)	7.43 (50.01)	
Smoking status, *n* (in millions), %				<0.0001
Never	98.54 (53.01)	93.91 (54.91)	4.63 (31.18)	
Former	47.05 (25.31)	42.17 (24.66)	4.88 (32.87)	
Current	40.29 (21.67)	34.95 (20.44)	5.34 (35.95)	
**Drinking status**, ***n*** **(in millions), %**				<0.0001
Never	20.11 (10.82)	18.75 (10.97)	1.35 (9.15)	
Former	29.51 (15.88)	25.42 (14.87)	4.08 (27.50)	
Current	136.26 (73.30)	126.85 (74.17)	9.41 (63.35)	
Body mass index (kg/m)^2^ Mean (SD)	28.825 (6.78)	28.688 (6.64)	30.404 (8.06)	<0.0001
Cardiovascular disease, *n* (in millions), %	16.72 (8.99)	13.07 (7.64)	3.64 (24.55)	<0.0001
Hyperlipidemia, *n* (in millions), %	129.56 (69.70)	117.81 (68.88)	11.75 (79.10)	<0.0001
Hypertension, *n* (in millions), %	70.71 (38.04)	62.68 (36.65)	8.03 (54.06)	<0.0001
Diabetes, *n* (in millions), %	24.06 (12.95)	20.60 (12.05)	3.46 (23.32)	<0.0001
DI-GM Mean (SD)	4.58(1.53)	4.59(1.53)	4.47 (1.51)	0.0055
**DI-GM group**, ***n*** **(in millions), %**				0.0543
0-3	45.17 (24.30)	41.28 (24.14)	3.88 (26.15)	
4	46.55 (25.04)	42.70 (24.97)	3.85 (25.93)	
5	44.62 (24.00)	41.06 (24.01)	3.55 (23.94)	
≥6	49.54 (26.65)	45.97 (26.88)	3.56 (23.98)	
Beneficial to gut microbiota, Median (IQR)	2.00 (1.00, 3.00)	2.00 (1.00, 3.00)	2.00 (1.00, 3.00)	0.0011
Unfavorable to gut microbiota, Median (IQR)	2.00 (2.00, 3.00)	2.00 (2.00, 3.00)	2.00 (2.00, 3.00)	0.7104

SD, standard deviation; IQR, interquartile range; DI-GM, dietary index for gut microbiota; COPD: chronic obstructive pulmonary disease; NHANES, National Health and Nutrition Examination Survey.

### 3.2 Association between DI-GM and COPD

In multivariable logistic regression analyses, DI-GM expressed as a continuous variable was negatively correlated with an increased prevalence of COPD (OR, 0.95; 95% CI, 0.92–0.99; *P* = 0.006; Table 2, model 1). After adjusting for the potential confounders, each one-point increase in DI-GM was linked to a 4% reduction in the risk of COPD (OR, 0.96; 95% CI, 0.92–0.99; *P* = 0.016; Table 2, model 4). The association remained consistent when the DI-GM was converted into a categorical variable. In the fully adjusted model, individuals with DI-GM ≥ 6 showed a significant negative association with the risk of COPD (OR = 0.84; 95% CI, 0.73–0.98; *P* = 0.023; Table 2, model 4). Additionally, as the beneficial to gut microbiota increased, the prevalence of COPD significantly decreased (OR = 0.95; 95% CI, 0.91–1.00; *P* = 0.030; Table 2, model 4), whereas no significant association was observed between the unfavorable to gut microbiota and COPD.

**Table 2:** Association between DI-GM and COPD of the NHANES 1999–2018 participants.

	**Model 1**		**Model 2**		**Model 3**		**Model 4**	
	**OR (95%CI)**	***p*** **-value**	**OR (95%CI)**	***p*** **-value**	**OR (95%CI)**	***p*** **-value**	**OR (95%CI)**	***p*** **-value**
DI-GM	0.95 (0.92~0.99)	0.006	0.89 (0.86~0.92)	<0.001	0.95 (0.91~0.98)	0.006	0.96 (0.92~0.99)	0.016
DI-GM group								
0–3	1 (Ref)		1 (Ref)		1 (Ref)		1 (Ref)	
4	0.96 (0.84~1.09)	0.531	0.88 (0.77~1.01)	0.061	0.94 (0.82~1.07)	0.357	0.95 (0.83~1.09)	0.500
5	0.92 (0.79~1.07)	0.271	0.77 (0.66~0.89)	<0.001	0.88 (0.76~1.02)	0.082	0.89 (0.77~1.03)	0.113
≥6	0.82 (0.71~0.95)	0.010	0.62 (0.53~0.72)	<0.001	0.82 (0.71~0.95)	0.009	0.84 (0.73~0.98)	0.023
Trend test		0.011		<0.001		0.008		0.020
Beneficial to gut microbiota	0.93 (0.89~0.97)	<0.001	0.86 (0.82~0.90)	<0.001	0.94 (0.90~0.99)	0.009	0.95 (0.91~1.00)	0.030
Unfavorable to gut microbiota	0.99 (0.95~1.04)	0.761	0.94 (0.90~0.99)	0.019	0.97 (0.92~1.02)	0.224	0.97 (0.92~1.02)	0.257

DI-GM, the dietary index for gut microbiota; COPD, chronic obstructive pulmonary disease; NHANES, National Health and Nutrition Examination Survey; OR, Odd Ratio; CI, Confidence interval.

Model 1: unadjusted; Model 2: adjusted for age, sex, race; Model 3: adjusted for Model 2+ marital status, poverty income ratio, educational level, smoking status, drinking status, body mass index; Model 4: adjusted for Model 3+ cardiovascular disease, hyperlipidemia, hypertension, diabetes. The DI-GM ranges from 0–13 (including beneficial to gut microbiota [ranges from 0–9] and unfavorable to gut microbiota [ranges from 0–4]) and grouped according to 0–3, 4, 5, and ≥ 6.

### 3.3 Subgroup analyses

**Figure 2:**
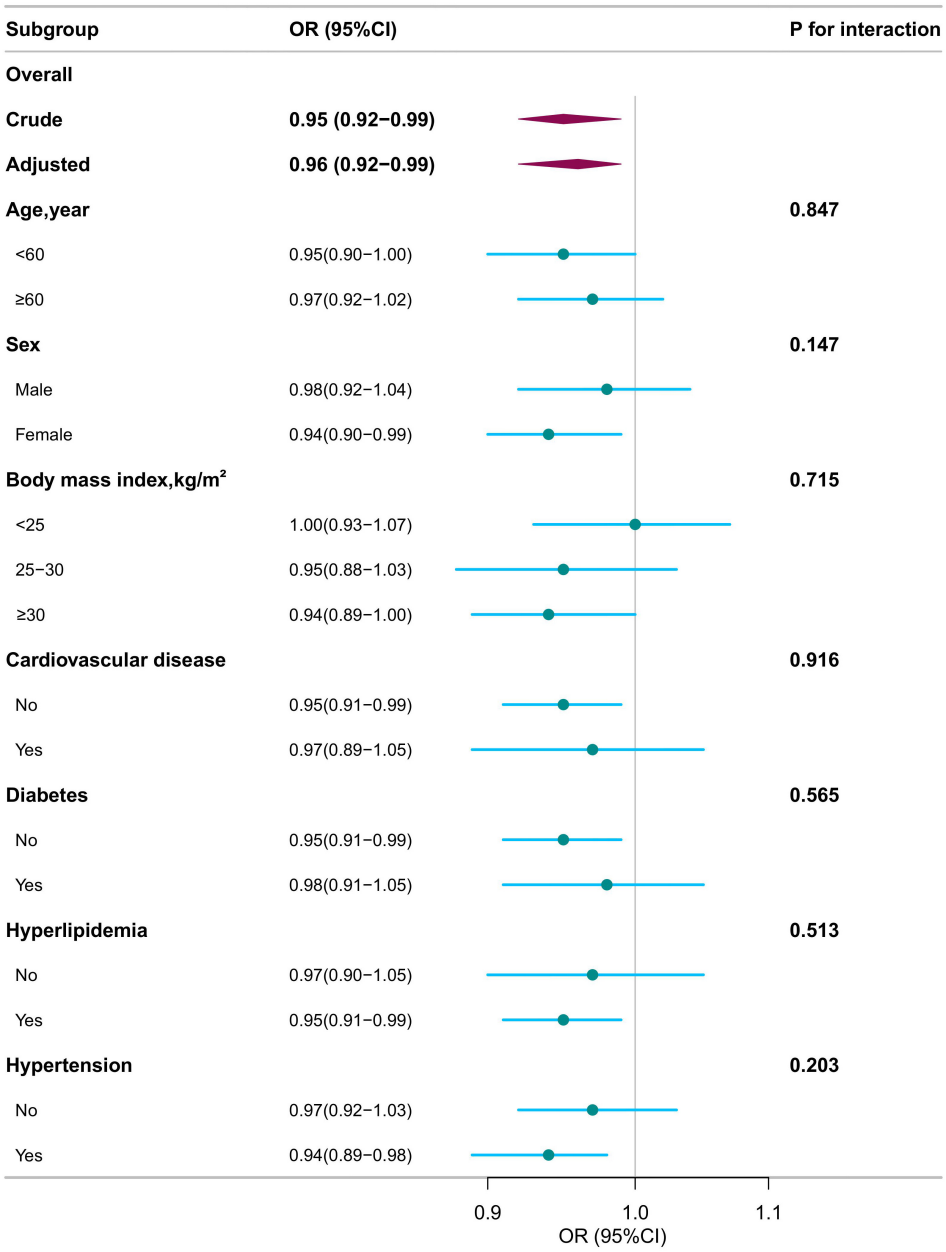
Subgroup analyses for the association of dietary index for gut microbiota and chronic obstructive pulmonary disease. OR, odds ratio; CI, confidence interval. Except for the stratification component itself, each stratification factor was adjusted for all other variables (age, sex, race, marital status, poverty income ratio, educational level, smoking status, drinking status, body mass index, cardiovascular disease, hyperlipidemia, hypertension, diabetes).

Stratified analysis was conducted in several subgroups to evaluate potential effect modifications on the association between DI-GM and COPD (Figure 2 and Supplementary Table 2). No significant interactions were observed in any subgroups after stratification by sex, age (20–60 vs. ≥60 year), BMI (< 25 vs. 25–30 or ≥30 kg/m^2^), CVD, diabetes, hyperlipidemia, and hypertension (all *P* for interaction > 0.05) (Figure 2).

### 3.4 Sensitivity analysis

In the sensitivity analysis, unweighted logistic regression analysis was performed. The DI-GM was found to be significantly associated with COPD after adjusting for all confounders (OR = 0.96; 95% CI, 0.94–0.99; *P* = 0.004; Table 2). Additionally, multiple interpolation was used to address missing data. Among the 47,328 participants included, 3,677 (7.7%) had COPD. The association between DI-GM and COPD remained statistically significant. After adjusting for confounders, an elevation in DI-GM was linked to a 4% reduction in the incidence of COPD (95% CI, 0.94–0.99; *P* = 0.004; Table 3). The adjusted OR for individuals with DI-GM ≥ 6 was 0.86 (95% CI, 0.77–0.97, *P* = 0.011), compared to those with lower DI-GM (Table 3).

**Table 3:** Sensitivity analysis.

**Analysis**	**Total**	**Event (%)**	**Crude model**		**Adjusted model**	
			**OR (95%CI)**	**p-value**	**OR (95%CI)**	**p-value**
**Unweighted logistic regression analysis**						
DI-GM	40018	3177 (7.9)	0.97 (0.94~0.99)	0.009	0.96 (0.94~0.99)	0.004
DI-GM group						
0–3	10358	849 (8.2)	1 (Ref)		1 (Ref)	
4	10298	872 (8.5)	1.04 (0.94~1.14)	0.481	1.02 (0.92~1.13)	0.711
5	9606	734 (7.6)	0.93 (0.84~1.03)	0.147	0.88 (0.79~0.98)	0.024
≥6	9756	722 (7.4)	0.90 (0.81~0.99)	0.036	0.87 (0.78~0.97)	0.013
Trend test				0.008		0.002
Beneficial to gut microbiota	40018	3177 (7.9)	0.96 (0.93~0.99)	0.004	0.96 (0.93~1.00)	0.031
Unfavorable to gut microbiota	40018	3177 (7.9)	0.99 (0.96~1.03)	0.648	0.97 (0.93~1.01)	0.101
**Logistic regression analysis after multiple interpolation for missing covariates**						
DI-GM	47328	3677 (7.7)	0.97 (0.94~0.99)	0.003	0.96 (0.94~0.99)	0.003
DI-GM group						
0–3	12218	983 (26.7)	1 (Ref)		1 (Ref)	
4	12239	1008(27.4)	1.03 (0.94~1.12)	0.586	1.02 (0.92~1.13)	0.706
5	11331	862 (23.4)	0.94 (0.86~1.04)	0.211	0.88 (0.79~0.98)	0.022
≥6	11540	824 (22.4)	0.88 (0.80~0.97)	0.009	0.86 (0.77~0.97)	0.011
Beneficial to gut microbiota	47328	3677 (7.7)	0.96 (0.93~0.99)	0.005	0.96 (0.93~1.00)	0.031
Unfavorable to gut microbiota	47328	3677 (7.7)	0.98 (0.95~1.02)	0.299	0.97 (0.93~1.01)	0.101

DI-GM, dietary index for gut microbiota; COPD, chronic obstructive pulmonary disease; NHANES, National Health and Nutrition Examination Survey; OR, Odd Ratio; CI, Confidence interval.

The crude model was not adjusted for any covariates, while the adjusted model was adjusted for age, sex, race, marital status, poverty status, educational level, smoking status, drinking status, body mass index, cardiovascular disease, hyperlipidemia, hypertension, and diabetes. The DI-GM ranges from 0–13 (including beneficial to gut microbiota [ranges from 0–9] and unfavorable to gut microbiota [ranges from 0–4]) and grouped according to 0–3, 4, 5, and ≥ 6.

Based on model 4, we separately adjusted energy, protein, saturated fat, and dietary fiber intake. The result showed that the association between DI-GM and COPD remained stable (Supplementary Table 3). Furthermore, we evaluated the associations of HEI-2015, aMED, and DII with COPD prevalence. While all indices showed statistically significant associations with COPD in adjusted models (Supplementary Table 4), their area under the curve (AUC) from ROC analyses were low, indicating limited discriminative ability (Supplementary Figure 1).

## 4 Discussion

This nationally representative cross-sectional study revealed that higher DI-GM scores, being in the DI-GM≥6 group, and increased beneficial to gut microbiota were all significantly associated with a decreased prevalence of COPD. These findings were consistent in subgroups and sensitivity analysis.

The role of diet as a key environmental factor influencing human health and disease is well-established. A systematic review and meta-analysis of eight observational studies found that healthy dietary patterns were associated with a lower prevalence of COPD (pooled OR = 0.88; 95% CI, 0.82–0.94), while no such association was observed for unhealthy dietary patterns (21). A study in the UK population reported that a “prudent” dietary pattern, characterized by a high intake of fruit, vegetables, oily fish, and wholemeal cereals, was associated with improved lung function and a lower COPD prevalence (22). Similarly, an 11-year prospective study among Chinese adults suggested that a balanced diet that included ample amounts of soybeans, fresh fruit, poultry, meat, fish or seafood, eggs, and dairy products may reduce the risk of COPD (23). The DI-GM, which reflects changes in gut microbiota diversity induced by diet, highlights dietary patterns that are either beneficial or harmful to gut health. Consistent with previous research, our study demonstrated that a higher DI-GM and beneficial to gut microbiota were negatively associated with the risk of COPD.

The gut microbiota, which interacts with diet, plays a significant role in health outcomes. DI-GM reflected dietary-induced alterations in the diversity of gut microbiota. For example, in the DI-GM, fermented dairy and fiber are considered beneficial to gut microbiota. A randomized controlled trial (RCT) showed that diets rich in fermented foods promoted greater microbiota diversity and alleviated inflammation (24). In contrast, insufficient fiber intake has been linked to reduced microbial diversity (25). In the DI-GM, refined grains are classified as unfavorable to gut microbiota. Additionally, high fat-to-carbohydrate ratio diets have been shown to diminish the diversity of the gut microbiota (24).

Gut microbes interact with the lungs through the “gut–lung axis”, which has a significant impact on the onset and progression of COPD (5). Several studies have demonstrated differences in gut microbiota diversity between patients with COPD and healthy individuals (26). Li et al. (4) performed 16S rRNA gene sequencing analyses on stool samples from a cohort including healthy controls and COPD patients. Their findings revealed distinct differences in the gut microbiome of COPD patients, marked by altered microbial composition and diversity, with a Prevotella-dominated gut enterotype and reduced levels of short-chain fatty acids. Short-chain fatty acids have been shown to enhance lung function by modulating immune homeostasis and maintaining gut barrier integrity (27). In another study comparing 28 COPD patients to 29 healthy controls, several bacteria, including Streptococcus and various members of the Lachnospiraceae family, were associated with reduced lung function (26). Changes in DI-GM, influenced by dietary habits, affected the diversity of the gut microbiota and were associated with the prevalence of COPD.

In this study, subgroup analyses indicated that the association between DI-GM and COPD was consistent across different genders, ages, income groups, or educational levels. These findings suggest that diet patterns associated with the gut microbiota may have broad relevance to reducing the risk of COPD. However, previous studies have shown that different genders, ages, and socioeconomic statuses may influence the intake of dietary nutrients (28, 29). Given this, it may still be necessary to develop targeted dietary recommendations to increase the intake of foods beneficial to the microbiota.

The study has several limitations. First, its cross-sectional design prevents the establishment of a direct causal link between DI-GM and COPD. Additional longitudinal studies and RCTs are required to confirm the causal relationship. Second, as with most observational studies, this research cannot exclude potential confounding factors from measurement error or unmeasured variables. Third, although the original DI-GM was derived from 14 food items, green tea was excluded from the analysis because specific types of tea were unavailable in the NHANES 24-h dietary recall data. That may affect its completeness and comparability across individuals or populations. Finally, DI-GM scores were based on 24-h dietary recall data, respectively, which may introduce recall bias. However, previous research has also shown that 24-h dietary recalls explained more variance in short-term energy and protein intake biomarkers than food frequency questionnaires (30). Additionally, the diagnosis of COPD depended on self-report, and some important variables including lung function measures were missing, which could introduce bias.

## 5 Conclusions

In conclusion, our findings suggest that DI-GM had a significant negative association with the prevalence of COPD. However, further research is needed to validate the DI-GM in clinical populations and to assess the causal and mechanistic pathways linking diet, gut microbiota, and lung health. Longitudinal studies and randomized controlled trials will be essential to determine whether dietary modification can improve microbiota composition and respiratory outcomes in at-risk or affected individuals.

## Data Availability

Publicly available datasets were analyzed in this study. This data can be found here: https://www.cdc.gov/nchs/nhanes/index.html.

## Supplementary material

Supplementary Figure 1 The Area Under the Receiver Operating Characteristic Curve (AUC-ROC). DI-GM, dietary index for gut microbiota; aMED, alternative Mediterranean Diet Score; DII, Dietary Inflammatory Index; HEI-2015, the Healthy Eating Index. **(A)** The model including DI-GM and DII; **(B)** the model including DI-GM and aMED; **(C)** the model including DI-GM and HEI-2015.

Supplementary Table 1 Components of the DI-GM.

Supplementary Table 2 Subgroup analyses for the association of dietary index for gut microbiota and chronic obstructive pulmonary disease.

Supplementary Table 3 Sensitivity Analysis.

Supplementary Table 4 The association between DI-GM, aMED, DII, HEI-2015 and COPD.
