# Supplementary figures and images for: Association of the dietary index for gut microbiota and chronic obstructive pulmonary disease: a cross-sectional study
Source: Front Nutr. 2025 Aug 26;12:1596424. doi: 10.3389/fnut.2025.1596424 (PMC12418446; doi:10.3389/fnut.2025.1596424)

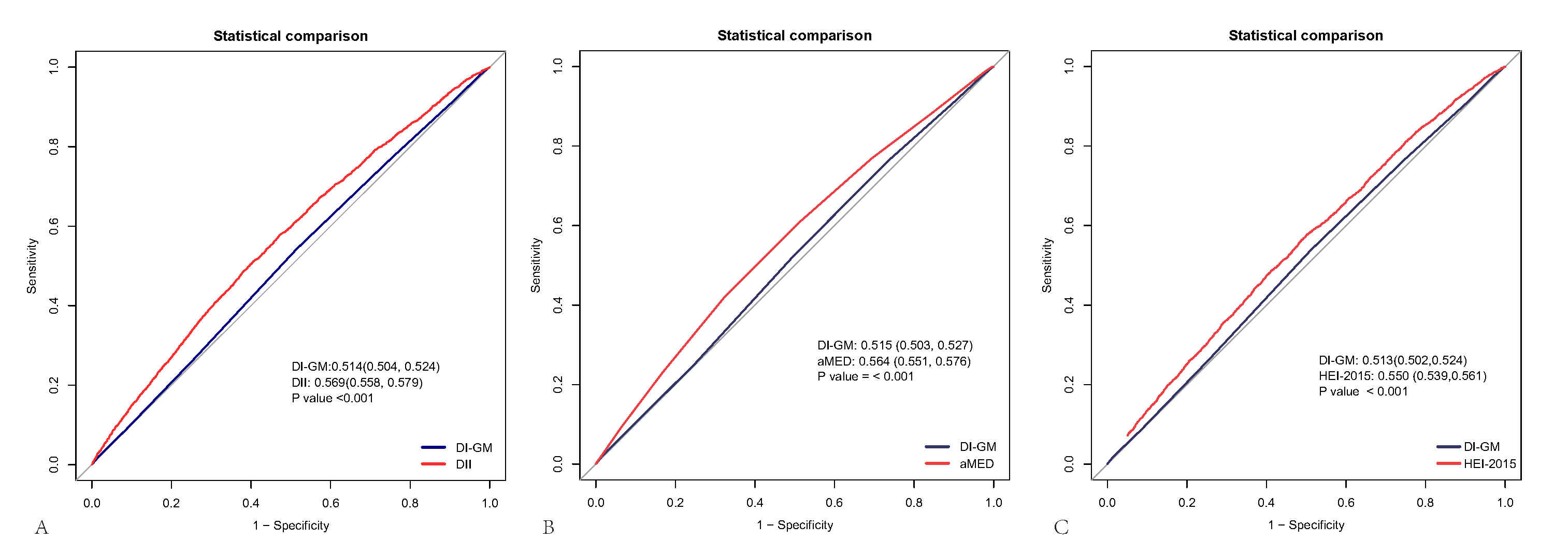

Supplement: Supplementary Figure 1 — The Area Under the Receiver Operating Characteristic Curve (AUC-ROC). DI-GM, dietary index for gut microbiota; aMED, alternative Mediterranean Diet Score; DII, Dietary Inflammatory Index; HEI-2015, the Healthy Eating Index. (A) The model including DI-GM and DII; (B) the model including DI-GM and aMED; (C) the model including DI-GM and HEI-2015. [file Image_1.jpeg]
