# Supplementary material for: Association of the dietary index for gut microbiota and chronic obstructive pulmonary disease: a cross-sectional study
Source: Front Nutr. 2025 Aug 26;12:1596424. doi: 10.3389/fnut.2025.1596424 (PMC12418446; doi:10.3389/fnut.2025.1596424)
Supplement: Supplementary Table 1 — Components of the DI-GM. [file Table_1.docx]

Supplementary table 1 components of the DI-GM^a^.

| Component | Included Foods within the Component | scoring |
| --- | --- | --- |
| Beneficial to gut microbiota | | For each component, a score of 1 if consumption at or above the sex-specific median, else 0 |
| Avocados | Avocados |  |
| Broccoli | Broccoli |  |
| Chickpea | Chickpeas |  |
| Coffee | Coffee |  |
| Cranberries | Cranberries |  |
| Fermented dairy | Yogurt, cheese, kefir, sour cream, buttermilk |  |
| Fiber | Not applicable |  |
| Green tea | Green tea |  |
| Soybean | Soy products--Soy milk, Tofu |  |
| Whole grains | Grains defined as whole grains, containing the entire grain kernel—the bran, germ, and endosperm |  |
| Unfavorable to gut microbiota | |  |
| High-fat diet (% energy) | Not applicable | 0 if consumption at or above 40% energy from fat, else 1  For each remaining component, a score of  0 if consumption at or above the sex-specific median, else 1 |
| Processed meat | Frankfurters, sausages, corned beef, and luncheon meat that are made from beef, pork, or poultry |  |
| Red meat | Beef, veal, pork, lamb, and game meat; excludes organ meat and cured meat |  |
| Refined grains | Refined grains that do not contain all of the components of the entire grain kernel |  |

Note: ^a^ Data sourced from Kase et al. (2024)
