# Supplementary material for: Association of the dietary index for gut microbiota and chronic obstructive pulmonary disease: a cross-sectional study
Source: Front Nutr. 2025 Aug 26;12:1596424. doi: 10.3389/fnut.2025.1596424 (PMC12418446; doi:10.3389/fnut.2025.1596424)
Supplement: Supplementary Table 2 — Subgroup analyses for the association of dietary index for gut microbiota and chronic obstructive pulmonary disease. [file Table_2.docx]

Supplementary table 2 subgroup analyses for the association of dietary index for gut microbiota and chronic obstructive pulmonary disease

|  | OR (95%CI) | P for interaction |
| --- | --- | --- |
| Poverty income ratio |  | 0.109 |
| ≤1.3 | 0.93 (0.89~0.97) |  |
| 1.3-3.5 | 0.99 (0.95~1.03) |  |
| >3.5 | 0.98 (0.93~1.03) |  |
| Educational level |  | 0.178 |
| Less than high school | 0.95 (0.9~1.00) |  |
| High school or equivalent | 0.99 (0.94~1.04) |  |
| Above high school | 0.96 (0.93~1.00) |  |
| Constipation status^*^ |  | 0.233 |
| Yes | 0.98 (0.94~1.03) |  |
| No | 0.84 (0.65~1.11) |  |

Note: Adjusted for age, sex, race, marital status, poverty status, educational level, smoking status, drinking status, body mass index, cardiovascular disease, hyperlipidemia, hypertension, and diabetes. *: the data from NHANES (2005-2010).
