# Supplementary material for: Association of the dietary index for gut microbiota and chronic obstructive pulmonary disease: a cross-sectional study
Source: Front Nutr. 2025 Aug 26;12:1596424. doi: 10.3389/fnut.2025.1596424 (PMC12418446; doi:10.3389/fnut.2025.1596424)
Supplement: Supplementary Table 3 — Sensitivity Analysis. [file Table_3.docx]

Supplementary table 3 Sensitivity Analysis.

| Analysis | Adjusted model | |
| --- | --- | --- |
|  | OR (95%CI) | p-value |
| Additional adjustment for energy intake | | |
| DI-GM | 0.96 (0.94~0.99) | 0.004 |
| DI-GM group |  |  |
| 0-3 | 1(Ref) |  |
| 4 | 1.02 (0.92~1.13) | 0.686 |
| 5 | 0.88 (0.79~0.98) | 0.025 |
| ≥6 | 0.87 (0.78~0.97) | 0.013 |
| Trend test |  | 0.002 |
| Additional adjustment for protein intake | | |
| DI-GM | 0.96 (0.94~0.99) | 0.004 |
| DI-GM group |  |  |
| 0-3 | 1(Ref) |  |
| 4 | 1.02 (0.92~1.13) | 0.727 |
| 5 | 0.88 (0.79~0.98) | 0.023 |
| ≥6 | 0.87 (0.78~0.97) | 0.013 |
| Trend test |  | 0.002 |
| Additional adjustment for carbohydrate intake | | |
| DI-GM | 0.96 (0.93~0.99) | 0.003 |
| DI-GM group |  |  |
| 0-3 | 1(Ref) |  |
| 4 | 1.02 (0.92~1.13) | 0.745 |
| 5 | 0.88 (0.79~0.98) | 0.019 |
| ≥6 | 0.86 (0.77~0.96) | 0.009 |
| Trend test |  | 0.001 |
| Additional adjustment for saturated fat intake | | |
| DI-GM | 0.96 (0.94~0.99) | 0.008 |
| DI-GM group |  |  |
| 0-3 | 1(Ref) |  |
| 4 | 1.02 (0.92~1.14) | 0.646 |
| 5 | 0.89 (0.8~0.99) | 0.032 |
| ≥6 | 0.88 (0.78~0.98) | 0.02 |
| Trend test |  | 0.003 |
| Additional adjustment for fiber intake |  |  |
| DI-GM | 0.98 (0.95~1.01) | 0.178 |
| DI-GM group |  |  |
| 0-3 | 1(Ref) |  |
| 4 | 1.04 (0.94~1.16) | 0.451 |
| 5 | 0.92 (0.82~1.02) | 0.127 |
| ≥6 | 0.93 (0.83~1.05) | 0.268 |
| Trend test |  | 0.087 |

Abbreviations: DI-GM, dietary index for gut microbiota; COPD: chronic obstructive pulmonary disease; NHANES, National Health and Nutrition Examination Survey; OR, Odd Ratio; CI, Confidence interval.

Note: Adjusted model was adjusted for age, sex, race, marital status, poverty status, educational level, smoking status, drinking status, body mass index, cardiovascular disease, hyperlipidemia, hypertension, and diabetes.
