# Supplementary material for: Association of the dietary index for gut microbiota and chronic obstructive pulmonary disease: a cross-sectional study
Source: Front Nutr. 2025 Aug 26;12:1596424. doi: 10.3389/fnut.2025.1596424 (PMC12418446; doi:10.3389/fnut.2025.1596424)
Supplement: Supplementary Table 4 — The association between DI-GM, aMED, DII, HEI-2015 and COPD. [file Table_4.docx]

Supplementary table 4 The association between DI-GM, aMED, DII, HEI-2015 and COPD

| Analysis | Crude model | | Adjusted model | |
| --- | --- | --- | --- | --- |
|  | OR (95%CI) | p-value | OR (95%CI) | p-value |
| DI-GM | 0.97 (0.94~0.99) | 0.009 | 0.96 (0.94~0.99) | 0.004 |
| aMED^*^ | 0.80 (0.76~0.84) | <0.001 | 0.86 (0.81~0.9) | <0.001 |
| DII | 1.14 (1.12~1.17) | <0.001 | 1.05 (1.03~1.07) | <0.001 |
| HEI-2015 | 0.99 (0.98~0.99) | <0.001 | 0.99 (0.99~0.99) | <0.001 |

Note: DI-GM, dietary index for gut microbiota; aMED, alternative Mediterranean Diet Score; DII, Dietary Inflammatory Index; HEI-2015, the Healthy Eating Index. The crude model was not adjusted for any covariates, while the adjusted model was adjusted for age, sex, race, marital status, poverty status, educational level, smoking status, drinking status, body mass index, cardiovascular disease, hyperlipidemia, hypertension, and diabetes.

^*^: the data from NHANES (2005-2018).
